# Expectations of efficient actions bias social perception: a pre-registered online replication

**DOI:** 10.1098/rsos.220889

**Published:** 2023-02-22

**Authors:** Katrina L. McDonough, Patric Bach

**Affiliations:** School of Psychology, University of Aberdeen, King's College, William Guild Building, Aberdeen AB24 3FX, UK

**Keywords:** action prediction, social perception, predictive processing, teleological reasoning, action efficiency, representational momentum

## Abstract

Humans take a teleological stance when observing others' actions, interpreting them as intentional and goal directed. In predictive processing accounts of social perception, this teleological stance would be mediated by a perceptual prediction of an ideal energy-efficient reference trajectory with which a rational actor would achieve their goals within the current environmental constraints. Hudson and colleagues (2018 *Proc. R. Soc. B* **285**, 20180638. (doi:10.1098/rspb.2018.0638)) tested this hypothesis in a series of experiments in which participants reported the perceived disappearance points of hands reaching for objects. They found that these judgements were biased towards the expected efficient reference trajectories. Observed straight reaches were reported higher when an obstacle needed to be overcome than if the path was clear. By contrast, unnecessarily high reaches over empty space were perceptually flattened. Moreover, these perceptual biases increased the more the environmental constraints and expected action trajectories were explicitly processed. These findings provide an important advance to our understanding of the mechanisms underlying social perception. The current replication tests the robustness of these findings and whether they uphold in an online setting.

## 1. Introduction

Our everyday social exchanges rely on our ability to imbue the actions of others with meaning and purpose [1–3]. We do not see other people's behaviours only in terms of the motions of their body parts, but in terms of the goals that the actors are trying to achieve and the obstacles they try to avoid (e.g. [4–6]). This ability to make sense of others' behaviours allows us to attribute mental states (goals, beliefs, etc.) to others, and generate expectations about what they might to do next, which is crucial for fluid social interactions [7,8]. By contrast, difficulties with reasoning about others’ behaviour characterizes several conditions associated with social deficits, such as autism and schizophrenia [9–12].

Conventional accounts have explained action understanding as a bottom-up process, in which observed actions are simply matched to the observer's own motor [13,14] or perceptual knowledge [15,16] and can thereby activate the relevant higher level knowledge about the goals and beliefs that may guide the actions. However, such approaches have difficulties resolving the many ambiguities inherent in social perception, such as many-to-one mapping that characterizes people's behaviour [4,17–20]. The same action can serve different purposes in different contexts (e.g. waving to greet versus waving to draw attention), and the same outcomes can be achieved by performing different actions (e.g. waving to greet versus an upwards nod of the head to greet). Therefore, the kinematics of an action alone cannot support a meaningful interpretation of another's behaviour (for a similar argument in vision, see [21]). Furthermore, a late, *post hoc* analysis of another's actions limits the ability to respond in timely manner, which may have costly consequences for fluent social interactions [8,22,23].

Recent predictive processing approaches to social perception (e.g. [4,24–28]) promise to provide a new solution to these issues. In these approaches, the motivations behind others' actions are not derived by a *post hoc* matching between an action and its meaning, but by a (pro-)active process of hypothesis testing and revision. Assumptions about others’ goals are derived from prior knowledge and translated into a perceptual ‘hypothesis’ of how a rational, intentional actor would efficiently realize them in the given environment. These expectations can then guide the perception of the others' actual behaviour, serving as a reference against which it can be compared. The resulting integration of sensory input with prior top-down expectations can explain not only how people can resolve even ambiguous social stimuli [29–31], and spontaneously predict how observed actions continue [32–34], but also why clearly mismatching behaviour stands out and prompts a revision to prior assumptions until they better explain the observed actions.

Recently, Hudson *et al*. [35–38] developed a novel experimental paradigm that was able to reveal the prior perceptual expectations that govern social perception and make them visible as a subtle perceptual bias towards what is expected (but not necessarily observed; for similar effects in memory, see [39,40]; outside of social perception, e.g. [41,42]). They hypothesized that the predictions people make of others' behaviour—and which are then tested against the actions that are observed—are governed by the principle of teleology: the assumption that people's behaviour is fundamentally goal directed, and that it will be actioned in the most rational and energy-efficient way [2,3,43]. This means that people should expect others to take the most direct path towards goals but to expend additional energy to circumvent obstacles along the way [3]. Hudson *et al*. [37] tested whether these expectations of efficient action could act as an ideal ‘reference’ trajectory for others' behaviour that biases its perception towards these expectations [35,36,38].

In a series of experiments, participants viewed short clips of an actor initiating a reach towards an object, which was sometimes obstructed by an obstacle. In half of the trials, the actor reached efficiently towards the object, taking a straight trajectory when the path was clear or a high, arched one to circumvent an obstacle in between. In the other half of the trials, however, the actor reached inefficiently: they would reach straight towards the goal object even though an obstacle obstructed this path or made an unnecessarily arched reach over empty space. The actor's hand suddenly disappeared midway through the action and participants indicated the last seen position of the actor's index finger on a touch-screen monitor. The results showed that participant's perceptual judgements were biased towards expectations of action efficiency. Thus, reaches with a straight trajectory were perceived to have reached higher when an obstacle had to be circumvented, in line with the expectation that the actor should have efficiently reached over it. Conversely, reaches with an arched trajectory were perceived to be lower when there was no obstacle to overcome, in line with the expectation that the actor would have reached efficiently straight towards the object. Furthermore, these perceptual biases were elicited spontaneously, when participants passively viewed the actions and reported their disappearance points but increased in magnitude (between-participants) when participants' attention was drawn to the presence or absence of an obstacle, or when they explicitly predicted the most efficient trajectory that the actor should take before the action started.

These results demonstrate, first, that people perceive the actions of others relative to how they would expect an intentional actor to behave, resulting in subtle perceptual biases towards the most efficient action in the given situation. They provide, second, a measure of this predictive influence on perception, in terms of the difference between what participants report to have seen and what was really presented. Third and finally, they showed that upweighting predictive information, by explicitly processing the environmental context or forming explicit expectations about the most efficient action, increases this predictive perceptual bias. These findings therefore provide an important advance to our understanding of the mechanisms underlying social perception, supporting the move away from traditional matching accounts, towards predictive processing theories of social perception (e.g. [4,24,28]), governed by an implicit assumption of efficient, goal-directed behaviour [2,3,43]. Given the potential importance of these findings and the general reproducibility problems that psychology experiments have faced in previous years [44–46], it is necessary to test the replicability and robustness of these findings, and to confirm that they hold up in an online setting. A successful online replication would further provide an avenue for future research to build upon this work regardless of in-laboratory restrictions (as previously experienced during the global COVID-19 pandemic), allow research to be conducted in a more timely manner compared with in-laboratory testing, and with the potential to reach much more diverse populations with greater ease than what is possible in the laboratory.

The current study replicates Hudson and colleagues’ study [37] in an online setting. To do so, some minor alterations to the methodology were necessary. These modifications were (i) the touch-screen response mode was replaced by a mouse-click for online compatibility, (ii) the verbal response mode used for explicit scene/prediction judgements was replaced by a button press for online compatibility, (iii) the number of trials was reduced by half to maintain the full engagement of the unsupervised participants, and (iv) the number of participants was increased to maintain a similar level of power given the reduced number of trials and potentially reduced effect sizes due the difference in response modality. If the original study is a true measure of the influence of action expectations on perception and provides valid evidence to support predictive processing accounts of social perception, then despite the minor methodological adaptations, we should replicate the original findings. Specifically, we expect that inefficient actions should be perceptually biased towards the expected efficient trajectory, such that inefficient straight reaches towards an obstacle will be perceived higher compared with efficient straight reaches when no obstacle is present, and inefficient arched reaches over an empty space will be perceived lower compared with efficient arched reaches over an obstacle. Moreover, these perceptual biases should be present when participants passively view the actions but should increase when they are asked to explicitly process the scene or state the expected trajectory.

## 2. Method

### 2.1. Participants

Participants were recruited from the Prolific participation pool in exchange for payment and were randomly assigned to one of three groups (No Task, Report Obstacle and Predict Trajectory). All participants reported normal/corrected-to-normal vision and gave informed consent. The study was approved by the University of Aberdeen's ethics board, in line with the Economic and Social Research Council (ESRC) and the Declaration of Helsinki. Sample size was determined by an *a priori* power analysis conducted on pilot data. Specifically, this was based on the effect size of the relevant interaction between the efficiency of the action (Reach Efficiency: efficient versus inefficient) and the trajectory of the action (Reach Trajectory: straight versus arched), in the group in which participants passively viewed the actions and where the smallest effect size was expected (No Task group, Cohen's *d* = 0.42). This revealed that a sample size of 50 participants per group, with a total sample size of 150 participants, would provide 90% power (alpha = 0.05) in the predicted direction. When accounting for potential participant exclusion based on pre-registered criteria (see Data processing), for which the original study and pilot studies have yielded 8–16% participant exclusions, the current experiment would still achieve 80% power if at least 37 participants (exclusion rate of 26%) remain in each group.

### 2.2. Apparatus and stimuli

Inquisit (Millisecond) software was used to program and host the experiment online via the Prolific participant recruitment platform.

**Figure 1.**
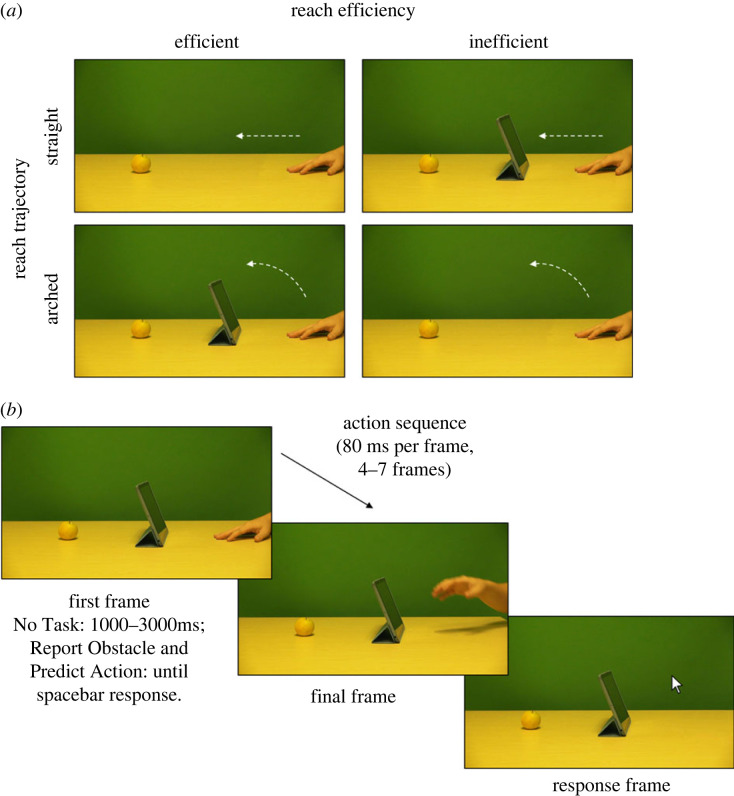
Stimulus conditions and trial sequence. The four stimulus conditions are depicted in (*a*). The reach trajectory was either straight (top row) or arched (bottom row), depicted by the white arrows. The presence or absence of the obstacle made these trajectories either efficient (left column) or inefficient (right column). An example trial sequence is depicted in (*b*). Participants first saw the first frame of the action followed by the action sequence. The hand then disappeared and participants clicked its final location using the computer mouse.

Stimuli depicting an actor reaching for an object with either an efficient or inefficient trajectory were taken from the original study ([37], figure 1*a* for examples). The original stimuli were derived from videos of an actor's arm at rest to the right of the screen, which then initiated a reach towards a target object on the left of the screen. In the original experiment, five different target objects were used (an apple, a bottle, a packet of crisps, a glue stick or a stapler). The current, shorter study reduced this to four target objects, removing the bottle. The reaches were either directed straight for the target object (Straight/Efficient) or arched over an obstacle (Arched/Efficient). Again, the number of obstacles used was reduced from the four obstacles in the original study (an iPad, lamp, pencil holder or photo-frame) to three (photo-frame removed) in the current study. Each video clip was converted into 19 individual frames, where frame 1 depicted the initial rest position and frame 19 depicted the mid-point of the action. For each efficient action, an inefficient action sequence was created by digitally removing the obstacles from the Arched/Efficient videos (Arched/Inefficient), or by inserting the obstructing objects into the Straight/Efficient videos (Straight/Inefficient). The inefficient actions therefore depicted movement kinematics that were identical to the efficient actions, differing only by the presence or absence of the obstacle.

Response stimuli were created for each action by digitally removing the actor's arm from the scene, so that only the objects and background remained. This frame was presented immediately after each action sequence to give the impression of the hand disappearing from the scene and was used by participants to indicate its final location.

### 2.3. Procedure

Participants were randomly assigned to one of three group conditions (Group 1: No Task, Group 2: Report Obstacle, Group 3: Predict Trajectory) and completed a total of 96 trials, across two blocks, in which each type of trial was presented six times (2 reaches × 2 efficiencies × 4 sequence lengths). The total number of trials was reduced from the original study (160 trials) to shorten the duration of the experiment to a more manageable time for unsupervised participants to maintain full engagement. Power was maintained by increasing the number of participants, as suggested by the power calculations reported above.

The experimental procedure closely matched that of the original experiment and was only altered to enable online compatibility; any changes made are detailed throughout. An example trial sequence can be seen in figure 1*b*. At the start of each trial, participants saw the first frame of the action sequence as a static image, showing a hand resting on a table on the right side of the screen, with a target object visible on the left, and—in half of the trials—an obstacle in between. In the Predict Trajectory condition, participants had to mentally decide for themselves whether the actor should reach straight or make an arched reach, given the presence or absence of an obstructing object. In the Report Obstacle condition, participants had to decide whether there was an obstructing obstacle present, yes or no. Participants were instructed to press the spacebar with their non-dominant hand as soon as they made their decision. This spacebar response replaced a verbal response used in the original experiment to enable online compatibility and had been validated during a prior pilot stage using the same tasks. Finally, in the No Task condition, participants were not given any instructions about what to do before the action started. The trial continued automatically after a randomized delay between 1000 and 3000 ms, roughly matching the timing of participants' space bar presses in the other conditions.

The action sequence began 1000 ms after the spacebar response was registered (in the Predict Trajectory or Report Obstacle conditions), or the viewing interval terminated (in the No Task condition). During all action sequences, the mouse cursor was hidden to prevent participants from tracking the hand to enhance performance. This replaced a similar procedure in the original experiment where the spacebar was held during the action to prevent tracking with the participants finger prior to a touch-screen response. The action sequence was created by presenting every third frame of the action for 80 ms each. The length of each action was randomized between four to seven frames. For example, a seven-frame sequence would present the frames 1-4-7-10-13-16-19. The final frame was then immediately replaced with the response frame, which showed the same scene without the hand, creating the impression that the hand had disappeared. The mouse cursor appeared on this frame and participants used the mouse to click on the screen where they thought the final position of the tip of the observed index finger was, replacing the touch-screen response from the original studies. As soon as a response was registered, the next trial began. At the end of the experiment, participants were asked to provide feedback and to indicate any reasons to suggest that their data should not be used, for example, if they were distracted or interrupted, if they experienced any technical difficulties or if they did not understand the task instructions.

### 2.4. Data processing

Data exclusions were implemented in line with our pre-registered exclusion criteria (https://aspredicted.org/EWU_WZI) and closely matched that of the original experiment. For each participant, individual trials were excluded if the response times (RTs) were shorter than 200 ms or more than 3 s.d. above the sample mean. Participants with too few trials remaining (less than 50%) were removed. Participants were excluded if the distance between the real final coordinates and participant responses exceeded 3 s.d. of the sample mean or if the correlation between the real final coordinates and participant responses was less than 0.70 (instead of 3 s.d. from the mean in the original study, as online data has increased variability). Participants were also removed if they indicated any reasons in their feedback to suggest that they did not conduct the experiment appropriately.

### 2.5. Analyses

Analysis was conducted on the predictive perceptual bias. It was calculated by subtracting the real final coordinates of the tip of the index finger from the participant's selected coordinates on each trial. This resulted in separate ‘difference’ scores along the *X*- and *Y*-axis where positive *X* and *Y* scores represented a rightward and upward displacement, respectively, and negative *X* and *Y* scores represented a leftward and downward displacement respectively. A score of 0 on both axes indicated that the participant selected the real final position exactly. These difference scores were entered into a 2 × 2 × 3 mixed ANOVA for the *X*- and *Y*-axis separately, with Reach Trajectory (straight versus arched) and Reach Efficiency (efficient versus inefficient) as repeated-measures factors and Task (No Task, Report Obstacle, Predict Trajectory) as a between-subjects factor. To further investigate the robustness of these effects, we tested the Reach Trajectory × Reach Efficiency interaction separately for each group. Moreover, we performed between-subjects *t*-tests on the participants' interaction contrast values to assess how this interaction differed between the three groups. Please note that this analysis is mathematically equivalent to the pairwise three-way interactions of Reach Trajectory, Reach Efficiency and Task.

The predicted effect will be revealed as a two-way Reach Trajectory × Reach Efficiency interaction in the analysis of perceptual shifts along the *Y*-axis. Hands reaching inefficiently towards an obstacle will be perceived as higher than hands reaching efficiently, in line with the expectation that the actor would have reached over it. Conversely, hands reaching inefficiently over an empty space will be perceived lower than efficient reaches, in line with the expectation that the actor would have reached straight forwards. We further predict a three-way Reach Trajectory × Reach Efficiency × Task interaction, showing that the main two-way Reach Trajectory × Reach Efficiency interaction is present in the No Task condition and increases progressively when participants explicitly acknowledge the presence or absence of an obstructing obstacle (Report Obstacle condition) and when the most efficient trajectory is explicitly predicted (Predict Trajectory condition). We further predict that the interaction of Reach Trajectory and Reach Efficiency will be present in each of the three between-subject conditions separately. We do not make any specific predictions for perceptual shifts along the *X*-axis.

## 3. Results

One hundred and fifty participants took part in the experiment. One hundred and thirty-three participants (mean age = 26 years, s.d. = 5.4, 34 females, 99 males; No Task: *n* = 44, Report Obstacle: *n* = 49, Predict Trajectory: *n* = 40) were included in the analysis while 17 participants were excluded based on pre-registered exclusion criteria (see Data processing). Two participants were removed for having less than 50% trials remaining after RT trimming, five participants were removed because their localization judgements were more than 3 s.d. away from the sample mean, and 10 participants were removed for having low correlations (less than 0.70) between their responses and the real final positions.

### 3.1. *Y*-axis

Our predictions are tested by the analysis of perceptual deviations on the *Y*-axis. The analysis fully replicated the prior study [37]. It revealed a main effect of Reach Trajectory, *F*_1,130_ = 17.4, *p* < 0.001, $\eta_{p}^{2}=0.118$, with perceived disappearance points of the hand being displaced further upwards for arched (−2.97 px) reaches than for straight reaches (−5.85 px, *t*_132_ = 4.00, *p* < 0.001, *d* = 0.35). As predicted, it also showed the crucial interaction of Reach Trajectory and Reach Efficiency, *F*_1,130_ = 60.1, *p* < 0.001, $\eta_{p}^{2}=0.316$, revealing the biases induced by action efficiency expectations. Disappearance points for straight reaches were reported higher when the actions were inefficient (−4.61 px), due to the presence of an obstacle, than when the actions were efficient (−7.09 px; *t*_132_ = 5.43, *p* < 0.001, *d* = 0.47). Conversely, the perceived disappearance points for arched reaches were perceived to be lower for inefficient actions, when no obstacle was present, (−5.01 px) than for efficient actions (−0.094 px; *t*_132_ = 6.26, *p* < 0.001, *d* = 0.54).

**Figure 2.**
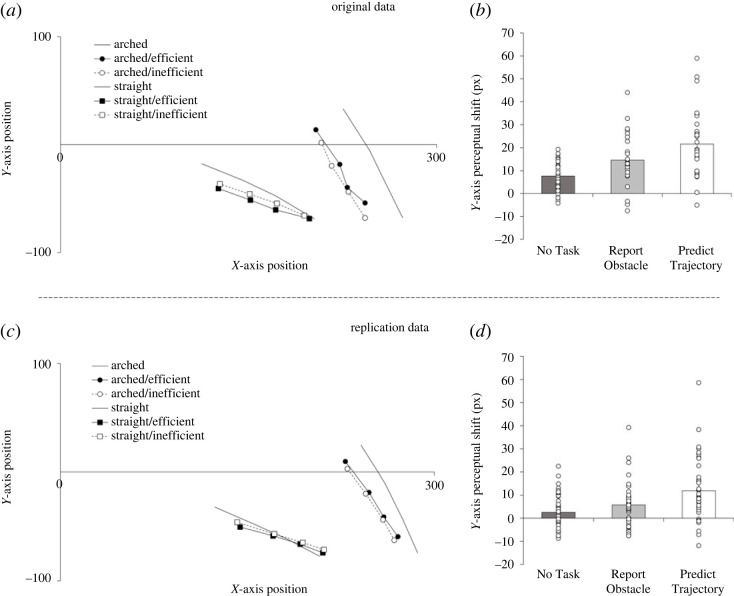
Data of the original study (*a*,*b*) and the current replication (*c*,*d*). (*a*,*c*) show localization responses of participants, split by reach trajectory (circles, arched reaches; squares, straight reaches) and efficiency (filled markers, efficient; empty markers, inefficient), and the actual disappearance of the tip of the hands' index finger. (*b*,*d*) show how size of the shift towards efficiency expectations varies by task instruction (from left to right: No Task, Report Obstacle and Predict Trajectory).

**Figure 3.**
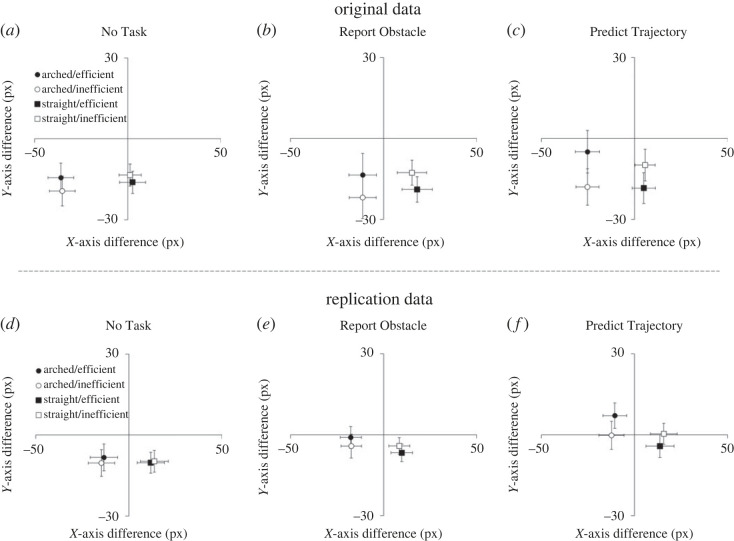
The Reach Trajectory × Reach Efficiency interactions for each task (No Task, Report Obstacle and Predict Trajectory) from the original study (*a*–*c*) and the current replication (*d*–*f*). These difference scores are the difference between the real final position of the hand (the centre of the plot where the axes cross) and participant's localization responses and represent the predictive perceptual bias.

Finally, the analysis also revealed the predicted three-way interaction between Reach Trajectory, Reach Efficiency and Task (*F*_1,130_ = 9.28, *p* < 0.001, $\eta_{p}^{2}=0.125$). The Reach Trajectory × Reach Efficiency interaction was larger in the Predict Trajectory condition than in the Report Obstacle condition (*t*_87_ = 2.59, *p* = 0.011, *d* = 0.55), which in turn was marginally larger than the No Task condition (*t*_91_ = 1.93, *p* = 0.057, *d* = 0.40; figure 2*d*). To demonstrate the robustness of the effect, we report here that the Reach Trajectory × Reach Efficiency interaction was evident for each condition separately (No Task: *F*_1,43_ = 5.48, *p* = 0.024, $\eta_{p}^{2}=0.113$; Report Obstacle: *F*_1,48_ = 22.7, *p* < 0.001, $\eta_{p}^{2}=0.321$; Predict Trajectory: (*F*_1,39_ = 30.4, *p* < 0.001, $\eta_{p}^{2}=0.438$; figure 3*d–f*).

As we had no further predictions, all other main effects and interactions should be treated as incidental unless they pass a (Bonferroni-)corrected threshold for hidden multiplicity in a three-factor ANOVA of *p* < 0.007 [47]. Only an interaction between Reach Trajectory and Task passed this threshold (*F*_1,39_ = 30.4, *p* < 0.001, $\eta_{p}^{2}=0.438$). The degree to which perceived disappearance points of the hand were displaced further upwards for arched reaches than for straight reaches was larger in the Predict Trajectory condition than in the No Task condition (*t*_82_ = 2.55, *p* = 0.013, *d* = 0.56), but there were no other differences between conditions (both *t*s < 1.48, both *p*s > 0.144). The main effect of Reach Efficiency (*F*_1,130_ = 6.56, *p* = 0.012, $\eta_{p}^{2}=0.048$) and the interaction between Reach Efficiency and Task did not pass the corrected significance threshold (*F*_1,130_ = 0.824, *p* = 0.441, $\eta_{p}^{2}=0.013$).

### 3.2. *X*-axis

As we had no predictions for the *X*-axis, all main effects and interactions should be treated as incidental unless they pass a (Bonferroni-)corrected threshold for hidden multiplicity in a three-factor ANOVA of *p* < 0.007 [47]. The analysis only revealed a main effect of Reach Trajectory, *F*_1,130_ = 698, *p* < 0.001, $\eta_{p}^{2}=0.843$, with perceived hand disappearance points being displaced further leftwards for arched (−14.6 px) reaches than for straight reaches (12.0 px, *t*_132_ = 26.7, *p* < 0.001, *d* = 2.6). This replicates the original study and most likely reflects the more right-displaced centre of gravity for straight arm configurations compared with arched configurations (e.g. [37,48]). There were no further main effects or interactions (all *F*s < 3.07, all *p*s > 0.054).

## 4. Discussion

The current study replicated the findings of Hudson *et al*. [37] in a pre-registered procedure adapted for online testing. The original study tested whether people observe the actions of others with reference to a perceptually represented ‘ideal’ reference trajectory, which describes how a rational, intentional actor would achieve their goals within the given environmental constraints (e.g. [2,3,43]). According to Bayesian/predictive processing accounts of social perception (e.g. [4,25–28]), such a prior expectation should guide the processing of the incoming visual information and create subtle perceptual biases towards the expected trajectory, which should increase the more explicitly the expectations are represented. The findings of both the previous study and the current replication support these predictions.

Participants watched short clips of an actor initiating either an efficient or inefficient reach towards an object and reported the hand's final position after it suddenly disappeared. We found, as in the original study [37], that perceptual reports of the last seen hand location of inefficient actions were shifted towards the expected, more efficient kinematics. When participants observed the actor's hand reaching straight towards an obstacle, they reported the hand to have disappeared higher compared with when there was no obstacle, in line with the expectation that an efficient actor would attempt to reach over the obstacle. Conversely, when participants observed an arched reach over empty space, they reported seeing the hand lower, in line with the expectation that the actor would have reached straight for the goal object. The data therefore support the hypothesis that prior expectations of how a rational actor would efficiently navigate the given environmental constraints were integrated with, and biased, the perception of the actor's actual behaviour towards these expectations.

The current study also replicated the results of the between-group task manipulation of the original study [37]. Participant's perceptual reports were predictively biased toward the efficient trajectory when they simply observed the actions without further task instructions (No Task condition). The biases increased, however, when participants were asked, before each action started, to explicitly report the environmental constraints that would affect the observed action (i.e. presence or absence of an obstacle; Report Obstacle condition). They increased further when participants were asked to explicitly predict the most efficient trajectory within these constraints (i.e. arched reach in case of an obstacle, straight reach when path is clear; Predict Trajectory condition). As in the original study, the perceptual biases therefore emerge spontaneously, but their contribution is weighted more strongly the more explicitly the relevant situational constraints and the resulting efficiency expectations are represented.

Together, these results fully confirm the findings of the original study and show that, during action observation, (i) people form predictions about the ideal trajectory that an intentional and rational actor would follow within given environmental constraints, that (ii) these expectations are integrated with the behaviour that is observed, so that it becomes subtly biased towards these more efficient trajectories, and (iii) that these biases increase the more strongly the prior efficiency expectations are weighted. This replication therefore shows that this general pattern of results is robust over changes in response device (mouse response versus touch screen) and the format with which participants made their pre-action judgements (key press versus verbal statements), as well as the changes in trial numbers between the original and the current study, even if effects sizes are generally lower in this online version compared with the original study (figure 2).

The current findings support predictive/Bayesian processing accounts of social perception (e.g. [4,24,28]), which assume that social perception is governed by a top-down process in which people project their current hypothesis about the other person's action onto the behaviour that is observed, resulting in subtle biases towards these expectations. The resulting integration of sensory input and prior expectation has been argued to underpin the attribution of meaning to (even ambiguous) behaviour of others, allows for an anticipatory planning of one's own behaviour in response, and the testing of one's prior expectations against what is observed so that they can remain aligned with reality (for extended argument, see [4]). Similar perceptual biases during social perception have now been observed in the memory for action events (e.g. [39,40]), to contribute to motion illusions (e.g. [49]), the perception of causality [50], and may underpin perceptual templates for common social interactions (e.g. chasing [51,52]). They may also govern our perception of non-social stimuli (e.g. gravity [53]; transitions between object states [41,42]), pointing towards a general role of predictive processing in perception, social and otherwise.

An open question is at what level of processing the efficiency expectations emerge. One possibility is that the expectations emerge from higher order ‘mentalistic’ knowledge about how an intentional actor would achieve their goals in different situations [2,3], which are translated into lower level expectations about which sensory action input would then be expected (e.g. [4,6]). Alternatively, efficiency expectations—and associated perceptual biases—could also be formed locally, within the higher order visual system itself (see [54,55]), without drawing on any higher order attributions of intentionality. Given that the people in our social world are (typically) intentional agents, efficient trajectories are statistically more likely than inefficient ones. Over development, the visual system could have ‘internalized’ these typical behaviour patterns, so that they can now be superimposed onto, and compared with, the action input that is received (for similar arguments about internalized physical forces, see [41,42,53]).

The data reported here and elsewhere suggest an influence from both sources. A top-down modulation is suggested by our finding that the perceptual biases increase substantially when participants are asked to explicitly process the environmental constraints and most efficient actions with them (i.e. the Report Obstacle and Predict Trajectory conditions). This suggests, at the very least, that more explicitly representing environmental features and action expectations increases their weighting in perceptual judgements, in line with Bayesian precision weighting theory [56,57]. Consistent with this interpretation, in another study in which people's stated goals (e.g. to ‘take’ or ‘leave’ an object) biased the perception of their subsequent actions, we found that making the goals cues more predictive about forthcoming actions (e.g. 75% versus 50%) did change the size of induced biases, but only when participants were also made explicitly aware of these changes [38]. Finally, we have found in another study that pre-action mental imagery (e.g. of a straight or arched reach, or of the presence or absence of an obstacle) allows people to *voluntarily* control their expectations about forthcoming actions, which induces highly similar perceptual biases as reported here [58]. These findings therefore imply some form of higher order ‘cognitive’ control of the expectations that guide social perceptual processing.

Other aspects of our findings suggest an independence of the measured biases from top-down guidance. In the No Task condition here, we found that efficiency expectations acted on action perception even when completely task irrelevant, suggesting that they are elicited by the observation of an intentional action itself, without cognitive control. A role of low-level visual-perceptual knowledge is also suggested by other studies, which show that relatively subtle (and probably implicit) manipulations that disrupt the biological motion profile of the actions also reduce the perceptual biases [59]. Moreover, in another study, we found that observation of reaches was biased towards an object that matched the currently formed hand grip (e.g. precision grip for small objects, whole hand grip for large objects). Like here, these biases were observed when the hand–object matches were task irrelevant. Surprisingly, their size did not increase when participants were asked to explicitly report the action's most likely goal, and they were dissociated from the explicit goal attributions people made on the same trials [30] (for similar evidence outside social perception, see [60,61]), suggesting an origin within the higher order visual system itself. It is also consistent with neuroimaging studies that show that higher order visual cortex and the superior temporal sulcus are sensitive to features such as animacy, intentionality and synchrony between interaction partners and the match between hand posture and available goal objects [62–64].

Together, therefore, the findings of the current and previous studies suggest that efficiency expectations can originate from the (higher order) visual system itself. However, these low-order expectations can at the very least be ‘tuned’ or become integrated with higher order action expectations from outside the visual system. Such an integration would make it possible for multiple perceptual hypotheses about others' behaviour, some originating from within visual system, some from without, to be tested in parallel and potentially revised.

## 5. Conclusion

The current study fully replicated the findings of Hudson *et al*. [37] in a modified procedure adapted to online testing, despite changes in response mode and trial numbers. The results confirm that people represent the actions of others relative to ideal ‘reference’ kinematics that describes how a fully cognizant, intentional actor would achieve their goals within the current environment constraint, which induces subtle biases towards these expectations, which increase the more explicitly the environmental constraints and action expectations are processed. The results support predictive processing/Bayesian processing theories of social perception (e.g. [4,24–28]) and link to similar perceptual expectations when observing changes in physical (non-social) scenes (e.g. gravity [53]; transitions between object states [41,42]). These findings therefore provide an important advance to our understanding of the underlying mechanisms of social perception. Furthermore, this work contributes to the efforts to tackle the general reproducibility crisis in psychology. Finally, the success of this replication with an online population provides a foundation for future work to continue this line of research free from the limitations of in-laboratory testing.

This article received results-blind in-principle acceptance (IPA) at Royal Society Open Science. Following IPA, the accepted Stage 1 version of the manuscript, not including results and discussion, was pre-registered on the OSF (https://osf.io/r4e5f/). This pre-registration was performed after data analysis.

## Data Availability

The data can be accessed here: https://osf.io/r4e5f/.
